# WHITE PANICLE3, a Novel Nucleus-Encoded Mitochondrial Protein, Is Essential for Proper Development and Maintenance of Chloroplasts and Mitochondria in Rice

**DOI:** 10.3389/fpls.2018.00762

**Published:** 2018-06-06

**Authors:** Hongchang Li, Guobiao Ji, Yun Wang, Qian Qian, Jichen Xu, Sodmergen, Guozhen Liu, Xianfeng Zhao, Mingsheng Chen, Wenxue Zhai, Dayong Li, Lihuang Zhu

**Affiliations:** ^1^State Key Laboratory of Plant Genomics and National Center for Plant Gene Research, Institute of Genetics and Developmental Biology, Chinese Academy of Sciences, Beijing, China; ^2^Shenzhen Institutes of Advanced Technology, Chinese Academy of Sciences, Shenzhen, China; ^3^China National Rice Research Institute, Hangzhou, China; ^4^School of Life Sciences, Peking University, Beijing, China; ^5^College of Life Sciences, Agricultural University of Hebei, Baoding, China

**Keywords:** *WHITE PANICLE3* (*WP3*), white stripe, white panicle, mitochondria, chloroplast, rice

## Abstract

Mitochondria and chloroplasts are interacting organelles that play important roles in plant development. In addition to a small number proteins encoded by their own genomes, the majority of mitochondrial and chloroplast proteins are encoded in the cell nucleus and imported into the organelle. As a consequence, coordination between mitochondria, chloroplasts, and the nucleus is of crucial importance to plant cells. Variegated mutants are chloroplast-defective mutants and are considered to be ideal models for studying the intercommunication between these organelles. Here, we report the isolation of *WHITE PANICLE3* (*WP3*), a nuclear gene involved in variegation, from a naturally occurring white panicle rice mutant. Disrupted expression of *WP3* in the mutant leads to severe developmental defects in both chloroplasts and mitochondria, and consequently causes the appearance of white-striped leaves and white panicles in the mutant plants. Further investigation showed that *WP3* encodes a protein most likely targeted to mitochondria and is specifically expressed in rice panicles. Interestingly, we demonstrate that the recessive white-panicle phenotype in the *wp3* mutant is inherited in a typical Mendelian manner, while the white-striped leaf phenotype in *wp3* is maternally inherited. Our data collectively suggest that the nucleus-encoded mitochondrial protein, WP3, plays an essential role in the regulation of chloroplast development in rice panicles by maintaining functional mitochondria. Therefore, the *wp3* mutant is an excellent model in which to explore the communication between the nucleus, mitochondria, and chloroplasts in plant cells.

## Introduction

In plant cells, chloroplasts and mitochondria are regarded as interdependent organelles due to their complex metabolic connections, such as metabolism, energy status, and reduction/ oxidation (redox) status (Raghavendra and Padmasree, 2003; van Lis and Atteia, 2004). Fine-level coordination between chloroplasts and mitochondria, namely chloroplast-mitochondria cross-talk, is therefore essential for the proper functioning and survival of plant cells. At present, genetic studies in various plant species have demonstrated the existence and importance of chloroplast-mitochondria cross-talk in the development of these organelles. First, functional disruption of mitochondria causes developmental defects in chloroplast. In potato, a reduction in the amount of P-protein, one of the subunits of mitochondrial glycine decarboxylase, leads to decreases in both photosynthetic and growth rates (Heineke et al., 2001). Similarly, defects in the barley mitochondrial glycine decarboxylase complex also result in chloroplast over-reduction and over-energization (Sabar et al., 2000). In turn, the damaged chloroplasts also affect mitochondrial development. In *Chlamydomonas reinhardtii*, delivery of chloroplast tRNAs to mitochondria has been demonstrated to be critical for protein synthesis in mitochondria (Bennoun and Delosme, 1999).

In addition to cross-talk between chloroplasts and mitochondria, nuclear genes are also largely involved in the development of these organelles (Pennazio, 2007; Pogson and Albrecht, 2011). To date, a number of nuclear genes that are involved in either chloroplast or mitochondrial developmental processes have been isolated. Most of these genes encode proteins that are targeted to these two organelles and function in multiple processes, including gene transcription, protein synthesis, enzyme activation, metabolite production, and electron transport chain maintenance (Barkan and Goldschmidt-Clermont, 2000; Woodson and Chory, 2008). Due to the critical importance of chloroplasts and mitochondria in bio-energetics and their roles in the synthesis of many cellular metabolites, tight coordination between the nucleus and these cytoplasmic organelles is essential for the survival and proper functioning of plant cells.

Variegated plants are defined as a category of mutants which display white or yellow sectors in plant green tissues such as leaves and stems. In particular, the variegation observed in monocotyledonous plants is sometimes referred to as striping (Sakamoto, 2003). A number of studies have demonstrated that most variegation is caused by chloroplast or mitochondrial defects, and the majority of these types of mutants arise from mutations in the nuclear genome (Aluru et al., 2006; Yu et al., 2007). Variegated mutants therefore provide ideal models for studying the intercommunication between the nucleus, chloroplasts, and mitochondria in plant cells. In the past two decades, a number of genes related to variegated leaf mutants have been identified, and most of these genes are located in the nucleus and encode proteins targeted to the chloroplasts or mitochondria. Thus, mutations within these genes generally lead to obvious developmental defects in chloroplasts or mitochondria. *Immutans* (*im*) is a variegated mutant isolated in Arabidopsis. Molecular studies show that the *IM* gene encodes a plastid terminal oxidase and functions as a terminal oxidase by transferring electrons from the plastoquinol pool to molecular oxygen. Mutation in *IM* gene results in the production of reactive oxygen and therefore causes the formation of photooxidized plastids in the white sectors of *im* (Wu et al., 1999; Carol and Kuntz, 2001; Rosso et al., 2009; Foudree et al., 2012). Two other variegated mutants that have been extensively investigated in Arabidopsis are *var1* (*variegation mutant 1*) and *var2* (*variegation mutant 2*). Studies on these two mutants indicate that both the VAR1 and VAR2 proteins play critical roles in chloroplast thylakoid membrane protein degradation (Sakamoto et al., 2003; Miura et al., 2007; Rosso et al., 2009). In addition to those genes that play essential roles in the chloroplast, several genes related to mitochondrial development have also been isolated from variegated mutants. Arabidopsis *CHM* (*chloroplast mutator*) is an example of one of these genes. Extensive studies show that *CHM* encodes a mitochondrion-targeted protein that is required for maintaining the integrity of mitochondrial DNA molecules (Sakamoto et al., 1996; Abdelnoor et al., 2003). Recently, Quesada et al. (2011) described an Arabidopsis mutant *rugosa2* (*rug2*) that displays pale green leaf sectors. The cloned *RUG2* gene was shown to encode a mitochondria and chloroplast dual-targeted protein that shows homology with metazoan mitochondrial transcription termination factors. Further study indicated that *RUG2* plays a role in modulating transcription of *RpoTp*, which encodes a chloroplast RNA polymerase, and thereby controls chloroplast development. Unlike the mutants mentioned above, in which the variegated phenotypes arise from mutations in the nuclear genome, mutations in chloroplast and mitochondrial genomes also frequently cause variegation. Maize non-chromosomal stripe (NCS) mutants are a series of typical mutants that result from specific DNA deletions in the mitochondrial genome. Deep explorations of these mutants showed that mitochondria DNA deletions in the NCS mutants leads to assembly defects in the mitochondrial respiratory complex (Karpova et al., 2002; Jiao et al., 2005). Nevertheless, the underlying mechanism through which dysfunctional mitochondria affect chloroplast development in the NCS mutants is unclear at present.

To study the mechanism of chloroplast development in grasses, we focused our attention on rice stripe mutants. In our previous study, we identified a naturally occurring rice mutant, which exhibits white panicles and white-striped leaves, in the progeny of an *indica*-*japonica* cross (Li et al., 2003). Because this mutant showed very similar characteristics to two previously characterized rice mutants, *wp1* (*white panicle1*) and *wp2* (*white panicle2*) (Sanchez and Khush, 1994, 2000), we named it *white panicle3* (*wp3*). Preliminary genetic analysis indicated that a mutation located on rice chromosome 1 may account for the white panicles in this mutant (Li et al., 2003). In our current study, we performed more detailed analyses of the mutant by using various approaches, including histology, electron microscopy, a map-based cloning strategy, and bioinformatics. Eventually, we showed that the *WP3* gene encodes a novel mitochondrial protein that is functionally disrupted in the *wp3* mutant by a retrotransposon insertion within the gene promoter. Histological and microscopic analyses demonstrated that the disrupted expression of *WP3* leads to severe mitochondrial and chloroplast developmental defects in the mutant panicles. Our study provides novel insights into the way that nuclear genes are involved in the regulation of chloroplast and mitochondrial development.

## Results

### Phenotypes of the Rice *white panicle3* (*wp3*) Mutant

**FIGURE 1:**
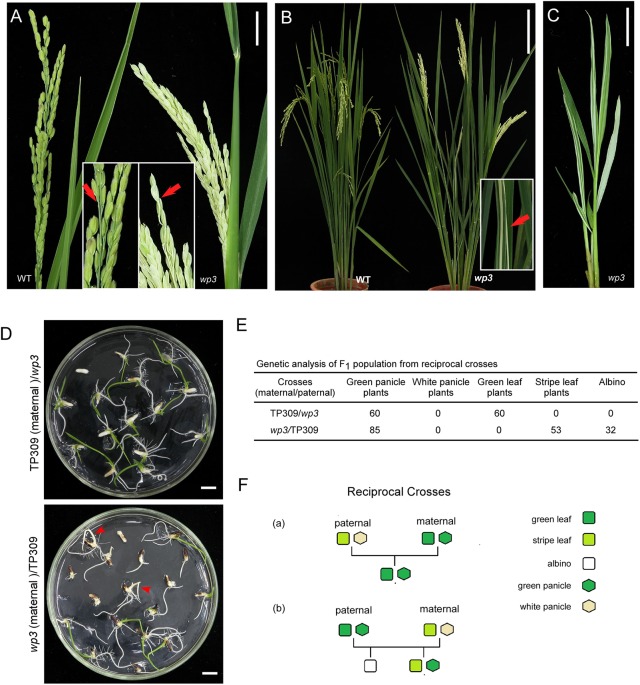
Phenotypic and genetic analysis of *wp3* mutant. **(A)** Representative images of wild-type (left) and *wp3* (right) panicles. Arrows in enlarged pictures indicated branches of panicle. Scale bars = 2 cm. **(B)** Representative images of leaves of wild type (left) and *wp3* (right) mutant. The white-stripe leaves in *wp3* plant were shown in enlarged picture. Arrow indicated the stripe leaves. Scale bars = 20 cm. **(C)** Image of *wp3* mutant at L5 stage. Scale bars = 2 cm. **(D)** Seedling F_1_ plants from reciprocal crosses between TP309 and *wp3* mutant. Arrows indicated the albinos. Scale bars = 1 cm. **(E)** Genetic analysis of F_1_ population from the reciprocal crosses. **(F)** Schematic overview of generations with various phenotypes from the reciprocal crosses.

We first performed detailed phenotypic analyses of rice *white panicle3* (*wp3*) mutant plants. As shown in **Figure 1A**, the mutant exhibits prominent milk-white colored panicles and rachises at the flowering stage, in contrast to the wild type plant in which the panicles and rachises are light green. Moreover, we noticed that the *wp3* mutant plants also have white-striped leaves, and it is interesting that the white stripes mostly appear on basal tiller leaves (**Figure 1B**). To fully explore the development of the striped-leaf character in the *wp3* mutant, we next examined *wp3* mutant plants at various growth stages. In the germination phase of the *wp3* mutant, most seedlings show white stripes on the leaves, while a portion of them (404 out of a total of 1448 seedlings) are albinos. All of the albino plants grew until the L3 stage before they died, whereas the other mutant plants survive but produce severe white-striped leaves prior to the L4 or L5 stage (**Figure 1C**). At the tillering stage, striped leaves are only occasionally observed at this late stage of development, mostly involving the first four or five tiller leaves of mutant plants. Furthermore, we observed that white sectors are not confined to restricted areas, but are widely distributed over the entire leaf surface, including the petiole. In addition, we found that the extent of variegation differs greatly among the individual mutant plants. Even in a single *wp3* plant, leaves with different degrees and patterns of variegation, including green, slightly striped, heavily striped, and totally white are observed.

Some chloroplast-defective mutant plants show significant sensitivity to changes in both temperature and light levels. To test whether the white-striped leaves in the *wp3* mutant are sensitive to these environmental changes, wild type and *wp3* mutant plants were grown under various temperatures (22, 28, and 32°C) or were exposed to different light intensities (500, 1000, and 1500 μmol quanta m^-2^s^-1^). Based on our examination, no dramatic phenotypic differences were observed in the *wp3* mutant plants compared to wild type as a result of these treatments, indicating that the generation of white stripes in the *wp3* mutant is independent of these environmental changes.

### In *wp3* Mutant Plants, White Panicles Are Determined by a Nuclear Gene, While White Stripes Are Maternally Inherited

Variegated mutants are mostly induced by chloroplast or mitochondrial defects, and variegated sectors in these mutants are therefore maternally inherited in their offspring (Abdelnoor et al., 2003). Our previous study demonstrated that the white-panicle phenotype in *wp3* mutant is inherited in a typical Mendelian manner and is determined by a single recessive nuclear gene (Li et al., 2003), but the inheritance pattern of the striped-leaf phenotype was not determined. To address this, reciprocal crosses between *wp3* and the wild type, TP309, were conducted and then subjected to genetic analysis. In F_1_ progeny from the cross in which TP309 was the maternal parent, all individual plants showed green leaves and panicles (*n* = 60). In contrast, F_1_ plants from the cross in which the *wp3* mutant was the maternal parent all had green panicles, but albinos (37.64%) and white-striped plants (62.36%) were detected (*n* = 85) during the seedling stage (**Figures 1D,E**). These data strongly suggest that white panicles and white-striped leaves in the *wp3* mutant are inherited by the progeny in two distinct ways. In contrast to the white-panicle phenotype that is inherited in a typical Mendelian fashion as a single recessive gene, the striped-leaf phenotype is maternally inherited (**Figure 1F**).

### Chloroplast Development Is Blocked in the *wp3* Mutant

**FIGURE 2:**
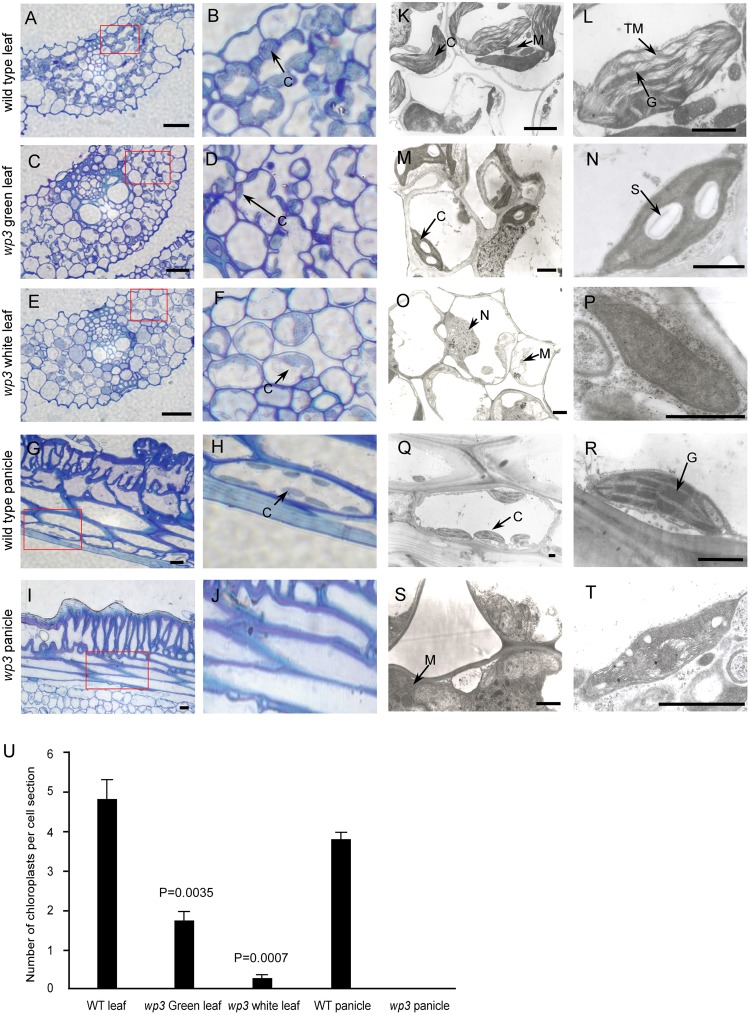
Chloroplast developmental defects in *wp3* mutant. **(A–J)** Histological images of cells from wild type and *wp3* mutant. **(A–F)** Showed the chloroplast in leaf cell; **(G–J)** showed the chloroplast in panicle cell. **(B,D,F,H,J)** Showed the enlarged images in red frames in **(A,C,E,G,I)**, respectively. C, chloroplast. Scale bars = 30 μm. **(K–T)** TEM images of chloroplast in wild type and *wp3* mutant. **(K–P)** Showed the chloroplast in leaf cell; **(Q–T)** showed the chloroplast in panicle cell. **(L,N,P,R,T)** Showed the single chloroplast. C, chloroplast; G, grana; M, mitochondria; N, nucleus; S, starch granule; TM, thylakoid membranes. Scale bars = 1 μm. **(U)** Quantification of chloroplast numbers in wild type and *wp3* mutant. The data from three biological replicates are shown as mean ± SD.

Considering that most variegated plants result from defective chloroplast development, we conducted a histological analysis to examine the chloroplasts in *wp3* mutant plants. Chloroplasts in the *wp3* mutant showed significant defects in both morphology and biogenesis as compared to those in wild type plants (**Figures 2A,B,G,H**). In the white stripes on *wp3* mutant leaves, chloroplast biogenesis seems to be completely blocked, because no chloroplasts with normal structures were observed in mesophyll cells (**Figures 2E,F**). A similar defect was also observed in panicle cells of *wp3* mutant plants (**Figures 2I,J**). Even in green leaves of *wp3* plants, most chloroplasts were smaller in size and had partially developed inner membranes (**Figures 2C,D**). Moreover, in *wp3* green leaf cells, the numbers of chloroplasts were markedly reduced compared with those in wild type plants. On average, the wild-type leaf had ∼4.82 chloroplasts per cell section, whereas <2 chloroplasts were observed in each *wp3* leaf cell section from within green sectors. More severely, almost no chloroplasts were found in cells from either white stripes or white panicles in the *wp3* mutant (**Figure 2U**).

We then collected leaf, palea/lemma, and branch tissues from *wp3* mutant plants for ultrastructure analysis using transmission electron microscopy. In wild type cells, chloroplasts were well developed with normally stacked grana and thylakoid membranes (**Figures 2K,L,Q,R** and **Supplementary Figure S1A**). In the *wp3* mutant, however, only a few smaller chloroplasts with less well developed grana were observed in green leaf cells (**Figures 2M,N**). Within cells in the white stripe sectors, some chloroplast-like organelles were observed, but all of them had completely lost the thylakoid membrane structures (**Figures 2O,P** and **Supplementary Figure S1B**), indicating that chloroplast development was significantly blocked in the mutant. We also observed that most chloroplasts in *wp3* mutant cells contained clear starch granules, suggesting that there are some chloroplast-related defects in starch metabolism in this mutant. All chloroplast abnormalities detected in white leaf stripe cells were also observed in white panicle cells (**Figures 2S,T**), suggesting that the *WP3* gene may play similar roles in chloroplast biogenesis in both the leaf and panicle.

We then investigated the chloroplast pigment constituents in the wild type and the *wp3* mutant. Strikingly, we detected a nearly 90 and 99% reduction in total carotenoids, including neoxanthin, violaxanthin, lutein, and β-carotene, in white panicles and white striped leaves of the *wp3* mutant, respectively, compared with that in the wild type. Moreover, sharp decreases in chlorophyll a and chlorophyll b were also found in the *wp3* mutant (**Supplementary Table S1**). These dramatic reductions in pigment content in the *wp3* mutant may be attributed to the severe chloroplast developmental defects caused by the mutation in *WP3*.

### Characterization of *WP3*

**FIGURE 3:**
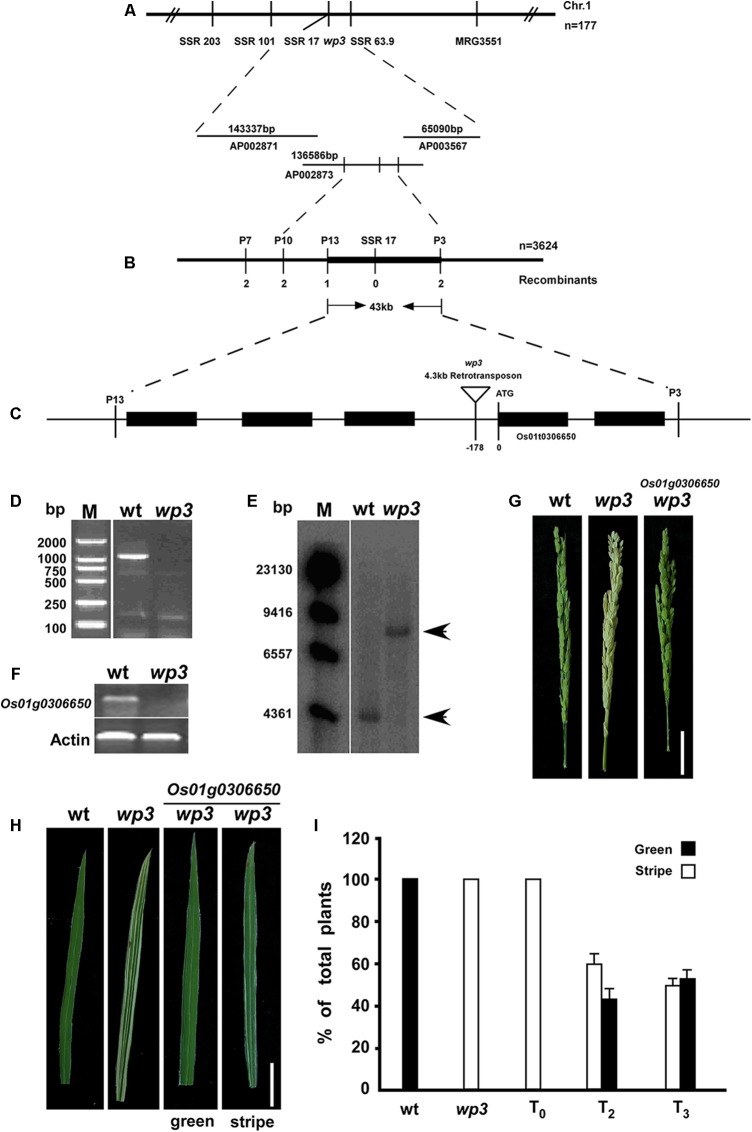
Map-based cloning of *WP3.* **(A)** Genetic mapping of *WP3* using SSR markers on the long arm of chromosome 1. BAC clones covering the corresponding region were shown. **(B)** Fine mapping of *WP3*. Number of recombinants was indicated below each marker. **(C)** Distributions of five predicted genes and schematic representation of *WP3* structure. The predicted translation start site (ATG) of *WP3* and site of retrotransposon insertion (inverted triangle) were indicated. **(D)** PCR amplification of *WP3* promoter region. M indicated the DNA ladder used. **(E)** Southern blotting in wild type and *wp3* plant. Genomic DNA from wild type and *wp3* plant was prepared, digested by *Eco*R I, transferred to membranes, and probed by a ^32^p-labeled DNA fragment containing *WP3* ORF. **(F)** RT-PCR amplification of *WP3* in wild type rice and *wp3* mutant. *Actin* was amplified as an internal control. **(G,H)** Representative images of panicle **(G)** and leaf **(H)** from wild-type, *wp3* and a transgenic *wp3* plant with expression of *WP3*. Scale bars = 5 cm. **(I)** Quantification of plants with white-stripe leaves in wild type, *wp3*, and *WP3* expressed *wp3* mutant at various generations.

The rice *WP3* gene was isolated by positional cloning based on segregation of the white/green panicle phenotype in an F_2_ population from a cross between the *wp3* mutant and a *japonica* cultivar, ‘*Zhi7*.’ We previously mapped the *WP3* locus to the long arm of chromosome 1 within a region of 3.1 centimorgans (cM) between two simple sequence repeat (SSR) marker loci, SSR101 and MRG3551 (**Figure 3A**). To clone the *WP3* gene, we performed fine-scale physical mapping using 3,624 homozygous white-panicle plants in another F_2_ population from the cross between *wp3* and ‘*Zhi7.*’ Several PCR-based markers, including SSRs, CAPS (cleaved amplified polymorphic sequences), and dCAPS (derived cleaved amplified polymorphic sequences), were developed by aligning *japonica* and *indica* genomic DNA sequences within the corresponding region and used in the fine mapping process. Finally, the *WP3* locus was narrowed down to a 43 kb genomic interval flanking CAPS marker locus P13 and dCAPS marker locus P3 (**Figure 3B**). Rice genomic sequences show that this region contains five annotated genes, which encode five putative proteins, including a transposon, a retrotransposon, an endoribonuclease E-like protein, and two additional hypothetical proteins (**Figure 3C**). Considering the potential relationship between the predicted functions of these genes and the mutant phenotypes observed in the *wp3* mutant, we ruled out the two transposon proteins and retained the remaining three genes as candidates for further testing.

In order to determine which of the three candidates was the *WP3* gene, the ORFs of these three genes were amplified from the two parental genomes and sequenced. Unexpectedly, no differences were identified between the allelic gene sequences from wild type and the *wp3* mutant. We then amplified the respective promoter regions of the three pairs of candidate genes from wild type and the *wp3* mutant. We found that no product was obtained using genomic DNA from the *wp3* mutant as a template to amplify the promoter region of one of the unknown genes (**Figure 3D**). We subsequently performed PCR amplification using various primer pairs that target the adjacent sequences of this region, but still no expected PCR products were obtained (data not shown). Based on these results, we speculated that a long sequence insertion could be present within this region in the *wp3* mutant genome.

Using a ^32^P-dCTP labeled PCR product of this gene from wild type rice as a probe, we next performed Southern blot analysis and detected a band that was 4 kb larger in the *wp3* mutant genome compared to the band found in the wild type rice genome (**Figure 3E**). Using TAIL-PCR and DNA sequencing, we obtained the terminal sequences of the insertion. Sequence analysis detected two typical LTR (Long Terminal Repeat) sequences in both ends of the insertion, indicating that the *wp3* mutant probably has a retrotransposon insertion in this region. Finally, we obtained the full sequence of the insertion using PCR and sequencing technologies (**Supplementary Figure S2**). Sequence analysis showed that this DNA insertion is a 4.3 kb *copia*-like retrotransposon located 178 bp upstream of the start codon of Os01g0306650 in the *wp3* mutant (Rice Annotation Project Database build4^1^). Semi-quantitative RT-PCR analysis suggested that this retrotransposon insertion in the promoter region of *Os01g0306650* led to reduced gene transcription in the *wp3* mutant (**Figure 3F**). To investigate the specificity of this retrotransposon insertion in the *wp3* mutant, we examined 22 rice varieties and found that only the *wp3* mutant, but not even one of the wild type rice varieties, contains this specific insertion (**Supplementary Figure S3**). Accordingly, we considered *Os01g0306650* the most likely candidate to be the *WP3* gene.

In order to confirm that *Os01g0306650* encodes *WP3*, we performed a complementation experiment. In brief, a 5.5 kb *Eco*RI-*Sal*I genomic DNA fragment containing the entire coding region, 3 kb upstream, and 2.3 kb downstream of the putative *WP3* gene was isolated from a BAC clone, inserted into the pCAMBIA1300 expression vector, and introduced into the genome of *wp3* plants via agrobacterium-mediated transformation. We obtained almost 50 independent transformants. Transformants carrying insertions of the DNA fragment that encodes *WP3* were identified using PCR. The expected amplification products for both *HygR* (*hygromycin B phosphotransferase*) and *WP3* were obtained from all 50 transformants, indicating that all transformants were positive for the transgene (**Supplementary Figure S4**). Subsequent phenotypic analysis showed that all transformants had green panicles, suggesting that *Os01g0306650* is *WP3* (**Figure 3G**). Unexpectedly, all transformants still exhibited white-striped leaves. Because chloroplasts are inherited from the mother plant, we thus speculate that the white stripes present on leaves of T_0_-generation transformant plants could be attributed to maternally inherited chloroplast defects from the mutant plants that were used as the receptor in the complementation test. If this is the case, the white stripes should be rescued in following generations from these transformants, since all transformants had green-colored panicles. Indeed, we observed that the number of plants with white-striped leaves was dramatically reduced in the subsequent T_2_ and T_3_ generations derived from these transformants (**Figure 3H**). While all T_0_ transformants exhibited a severe white-stripe phenotype, only about half of the T_2_- (77 out of 130) and T_3_-generation (66 out of 135) plants showed very slight striping on 1–2 leaves (**Figure 3I**). We followed the recovery of leaf color through the T_6_ generation of all transformants and found that the white-striped leaves were almost completely eliminated after the T_5_ generation. These data collectively support the notion that *WP3* is directly responsible for chloroplast development in rice panicles, while white-striped leaves on the *wp3* mutant are a byproduct of damaged chloroplasts in the panicles.

To further understand the roles of *WP3* in rice chloroplast development, *WP3* was over-expressed in a *japonica* rice variety, ‘Taipei 309,’ under control of the maize *UBI1* promoter. The T_0_ generation of *WP3* over-expressing plants did not show any differences with respect to leaf and panicle color. However, the T_1_-generation plants had much darker green leaves at the seedling stage compared with ‘Taipei 309’ plants (**Supplementary Figure S5**), suggesting that chloroplast development might be accelerated in the *WP3* over-expressing plants. This finding, combined with the fact that *wp3* mutant plants show severe chloroplast developmental defects, led us to speculate that *WP3* may have the capacity to improve chloroplast biogenesis.

### Sequence Analysis of the *WP3* Gene

**FIGURE 4:**
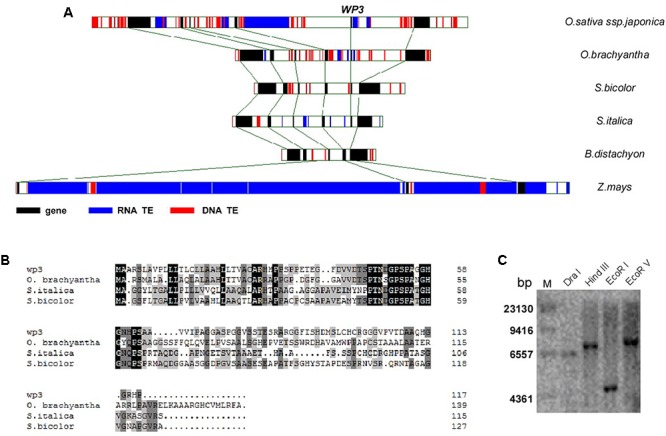
Genomic analysis of *WP3*. **(A)** Gene collinearity surrounding *WP3* in six monocot genomes was compared. **(B)** Alignment of amino acid sequences of *WP3* in rice and its orthologs in wild rice, sorghum, and millet. **(C)** Southern blot analysis of *WP3* in rice genome. Genomic DNA from a *japonica* rice, TP309, was prepared, digested by indicated restriction endonucleases, and subjected to southern blotting as in **Figure 3E**.

The *WP3* gene consists of a single open reading frame (GenBank Accession Number: JN794567) and encodes a putative protein containing 118 amino acid residues. Its N-terminal region, especially the first 22 amino acids, is enriched in hydrophobic amino acid residues and is therefore thought to be a possible signal peptide that mediates delivery to the cell membrane (**Figure 4B**). Southern blot analysis revealed that *WP3* is a single copy gene in the rice genome (**Figure 4C**). Unexpectedly, BLAST searches of the non-redundant protein database did not identify any orthologs of WP3. We speculate that this may be due to low levels of sequence homology of WP3 in different plant species. To further address whether the *WP3* gene is also present in other plant genomes, we carried out a gene collinearity analysis in all dicots and monocots with available genome sequences. We found that six monocot genomes showed similar gene collinearity surrounding the *WP3* locus; these included rice, the wild rice species *Oryza brachyantha*, sorghum, foxtail millet, *Brachypodium distachyon*, and maize (**Figure 4A**). Among these species, rice, wild rice, sorghum, and foxtail millet contain *WP3* homologs at the corresponding locus. Amino acid sequence alignment revealed that these four *WP3* homologs share obvious homology within their N-terminal domains (**Figure 4B**), indicating the functional significance of the N-terminus. Additionally, the conserved amino acid motif was used to construct a Hidden Markov Model (HMM) profile. The HMM profile was then used to search against the protein databases of *B. distachyon*, maize, Arabidopsis, and poplar. No *WP3* orthologs were identified in any of these species. Together, these data suggest that *WP3* is a gene that is unique to monocots, and that it is also restricted to some species of monocots.

### *WP3* Is Mainly Expressed in Rice Young Panicles

**FIGURE 5:**
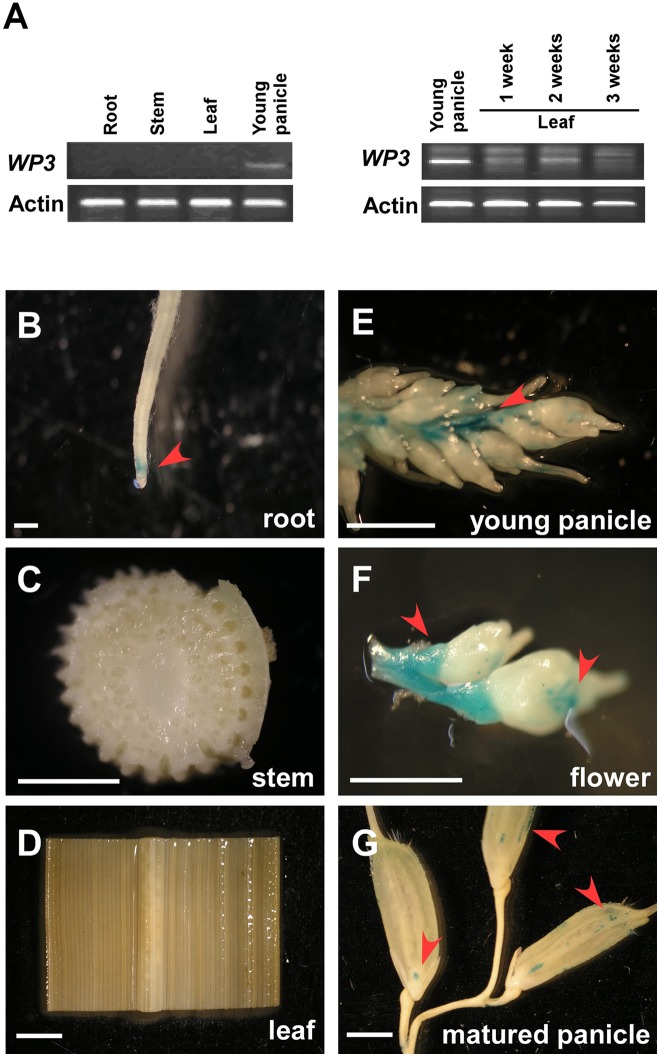
Expression patterns of *WP3*. **(A)** RT-PCR amplification of *WP3* in various rice tissues. *Actin* was used as a internal control. The experiments were performed in three biological replicates and representative results were shown. **(B–G)** *WP3* promoter driven GUS expression in rice root **(B)**, stem **(C)**, leaf **(D)**, young panicle **(E)**, flower **(F)**, and matured panicles **(G)**. Arrows indicated the expression of GUS. Scale bars = 0.2 cm.

To explore the expression pattern of *WP3* in rice, we performed reverse transcription PCR (RT-PCR) assays. Consistent with the fact that disrupted expression of *WP3* leads to white panicles, *WP3* transcripts were only detected in young panicles, but not in other tissues including roots, stems, and leaves (**Figure 5A**). We also examined the potential expression of WP3 in young leaves, but did not detect any expression of WP3 in these young tissues (**Figure 5A**). To further examine the expression of *WP3* in various tissues and at different developmental stages, a binary vector containing the *Escherichia coli uidA* gene (β-glucuronidase; GUS) driven by the *WP3* promoter (Pro*_WP3_*:GUS) was constructed and used to transform rice plants. We obtained 20 independent Pro*_WP3_*:GUS transgenic lines and used them to assay the promoter activation of *WP3*. We examined roots, leaves, stems, young panicles, flowers, and mature panicles for potential GUS expression. As expected, GUS signals were mainly observed in young panicles <5 mm and young flowers (**Figures 5E,F**), and no GUS staining signals were detected in rice leaves and stems (**Figures 5C,D**). However, in contrast with the previous result that *WP3* expression was only observed in young panicles by RT-PCR, a low level of GUS expression was also detected in roots and mature panicles (**Figures 5B,G**), indicating that *WP3* could also possibly play a role in the development of these tissues in addition to its major function in panicle genesis. Of note, the expression of *WP3* is only observed at the root tip but not in the entire root. Considering that the leaf equivalent of the root tip would be the very basal region of the leaf, *WP3* is also probably expressed at the very early step during leaf development.

### *WP3* Encodes a Mitochondrial Protein

**FIGURE 6:**
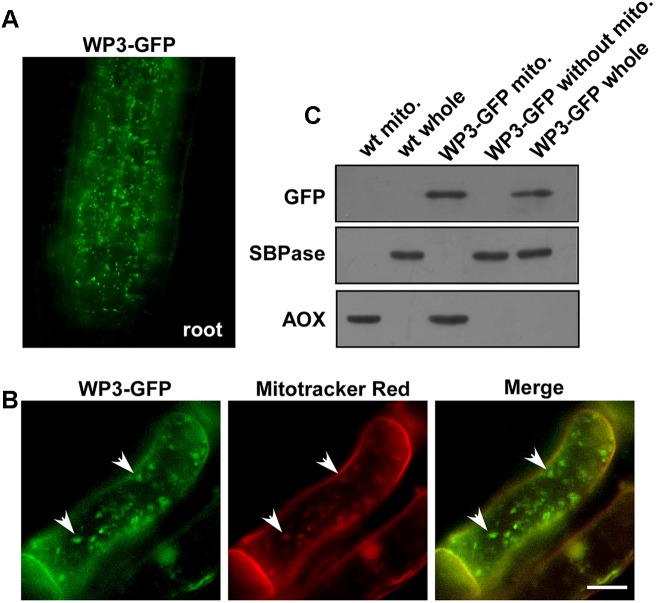
Mitochondrial localization of WP3. **(A)** Confocal microscopy image of wild-type rice stably expressing a WP3-GFP protein in root tip. **(B)** Co-localization of WP3-GFP and mitotracker red in rice root cells. Mitochondria were indicated by arrows. Scale bars = 5 μm. **(C)** Immunoblotting detection of WP3 in rice mitochondria fraction. Mitochondria fractions and total proteins from rice stably expressing WP3-GFP were prepared, separated in SDS-PAGE, transferred to membranes, and immunoblotted using antibodies as indicated. WT whole, whole leaf lysates from wild type plant; WT mito., mitochondrial fraction from wild-type plant; WP3-GFP whole, whole leaf lysates from WP3-GFP transgenic plant; WP3-GFP mito., mitochondrial fraction from WP3-GFP transgenic; WP3-GFP without mito., remained fractions after mitochondrial isolation from WP3-GFP transgenic plants.

As we mentioned above, disrupted expression of *WP3* resulted in severe chloroplast developmental defects; we next turned our attention to how *WP3* regulates chloroplast biogenesis in rice panicles. For this purpose, transgenic rice plants expressing the WP3-GFP fusion protein driven by the constitutive cauliflower mosaic virus 35S promoter, were generated and used to examine the cellular localization of WP3. Unexpectedly, the WP3-GFP protein did not show any co-localization with chloroplasts in rice leaves. Rather, the green fluorescence signals showed mitochondria-like localization patterns in root cells (**Figure 6A**). To test whether *WP3* encodes a mitochondrial protein, we performed staining using mitotracker red, which specifically stains mitochondria in eukaryotic cells. As indicated, WP3-GFP clearly co-localized with the red mitotracker red signals in root cells (**Figure 6B**). To further confirm these observations, mitochondrial fractions were isolated from cells of rice plants expressing WP3-GFP and subjected to immunoblotting analysis using antibodies as indicated in **Figure 6C**. As expected, we detected strong GFP signals in whole cell lysates and mitochondrial fractions from transgenic plants but not in control plants. Additionally, no signals were detected in the remaining fractions that did not contain mitochondria, further confirming the unique localization of WP3 in mitochondria. In this analysis, AOX and SBPase (sedoheptulose-1,7-bisphosphatase) were used as specific mitochondrial and chloroplastic fraction indicators, respectively. Both proteins were exclusively detected in the corresponding fractions, suggesting the high efficiency of the cell fractionation procedure (**Figure 6C**). Taken together, these data strongly support that the *WP3* gene encodes a mitochondrial protein.

### Disrupted Expression of *WP3* Leads to Developmental Defects in Mitochondria in the Rice *wp3* Mutant

**FIGURE 7:**
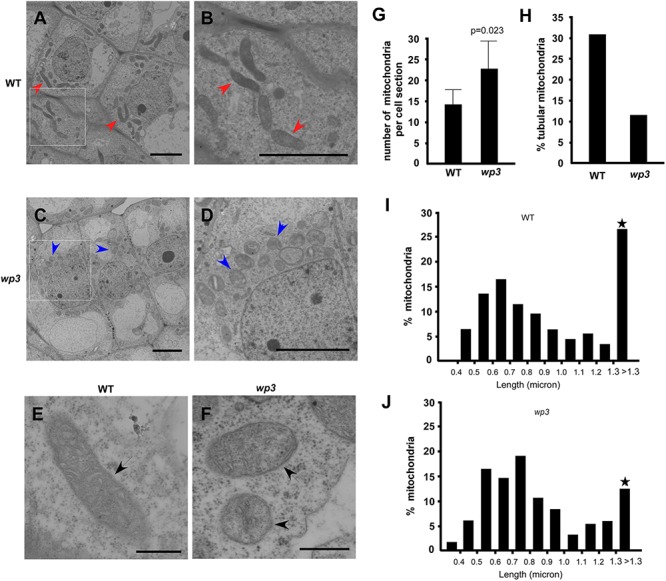
Mitochondria maturation is blocked/delayed in *wp3* mutant. **(A–F)** TEM images of mitochondria within wild-type **(A,B,E)** and *wp*3 ovary cells **(C,D,F)**. **(B,D)** Showed the enlarged images within frames of **(A,C)**. **(E,F)** Showed single mitochondria in wild type plant and *wp3* mutant, respectively. Arrows with red color in **(A,B)** indicated the elongated tubular mitochondria in wild-type cells; blue arrows in **(C,D)** indicated the round mitochondria in mutant cells; black arrows in **(E,F)** indicated the single mitochondria. **(G,H)** Quantification analysis of mitochondria numbers **(G)** and shapes **(H)** in wild type and *wp3* mutant. **(I,J)** Quantification of mitochondrial with various long axes in wild-type plant and *wp3* mutant cells. Stars indicated that the greatest difference was detected between wild type plant and *wp3* mutant.

To further explore the potential function of *WP3* in mitochondrial development, especially in rice panicles, we used electron microscopy (EM) to examine mitochondrial ultrastructure in wild type and *wp3* ovary cells. Compared with normally developed mitochondria within wild-type ovary cells, obvious defects in mitochondrial morphology and cristae organization were observed in ovary cells in the *wp3* mutant. First, most mitochondria in the mutant cells appeared to be spherical in shape, while the mitochondria in wild type cells presented multiple shapes, including spherical, elongated, and tubular (**Figures 7A–D**). Second, the observable mitochondria in *wp3* mutant ovary cells showed clearly disorganized cristae structures (**Figures 7E,F**). Additional quantification revealed that *wp3* mutant ovary cells contain disorganized, elongated mitochondria, despite the observation that they have more mitochondria than do wild type cells (**Figures 7G,H**). We further examined the size distribution of mitochondria in wild type and *wp3* cells. As shown in **Figures 7I,J**, no differences were detected between the wild type and mutant mitochondria with a long axis <1.3 μm, whereas the *wp3* cells showed clearly reduced percentages of mitochondria with a long axis >1.3 μm (**Figures 7I,J**), indicating that mitochondrial maturation in the *wp3* mutant is blocked and/or delayed.

**FIGURE 8:**
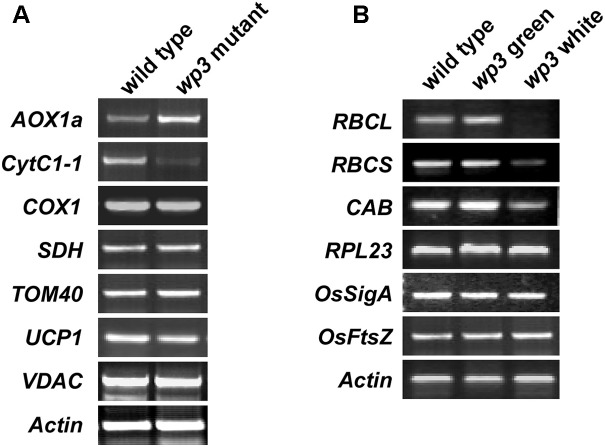
Semi-quantitative RT-PCR amplification of indicated genes related to mitochondria and chloroplasts development in wild type and *wp3* mutant. **(A)** 0.3–1 cm inflorescences were collected to prepare cDNA for amplification of mitochondria related gene. *Actin* was used for an internal control. *AOX1a*, alternative oxidase 1a; *CytC1-1*, cytochrome c1-1; *COX1*, cytochrome c oxidase subunit 1; *SDH*, succinate dehydrogenase; *TOM40*, translocase of outer mitochondrial membrane 40; *UCP1*, uncoupling protein 1; *VDAC*, voltage-dependent anion-selective channel. **(B)** RT-PCR analyses of chloroplast associated gene expression. Three-week-old plant leaves were collected for cDNA preparation. The white and green sectors in *wp3* stripe leaves were harvested separately. *RBCL*, the large subunit of chloroplast ribulose bisphosphate carboxylase; *RBCS*, the small subunit of chloroplast ribulose bisphosphate carboxylase; *RPL23*, 50S ribosome protein L23; *CAB*, the light-harvesting chlorophyll alb protein; *OsSigA*, plastid RNA polymerase sigma factor; *OsFtsZ*, plastid division protein FtsZ. The experiments were performed in three biological replicates and the representative results were shown.

To assess the effect of the *WP3* mutation on mitochondrial protein expression, we performed a series of semi-quantitative RT-PCR analyses. In total, seven mitochondria-related genes were selected for this experiment. Of these, four genes (*AOX1a*, *CytC1-1*, *SDH*, and *COX1*) encode proteins related to mitochondrial respiratory function, and three genes (*TOM40*, *VDAC*, and *UCP1*) encode channel proteins located on the mitochondrial membrane. As shown in **Figure 8A**, expression of two of these eight genes, *AOX1a* and *CytC1-1*, was obviously changed in *wp3* mutant panicles compared to wild type panicles. *CytC1-1* gene expression was clearly repressed, while *AOX1a* expression was markedly up-regulated in *wp3* young panicles (**Figure 8A**). In addition to *AOX1a* and *CytC1-1*, the other mitochondrial genes monitored in our study, including *COX1*, *SDH*, *TOM40*, *UCP1*, and *VDAC* showed the same expression levels in the wild type and *wp3* mutant plants. Since both AOX1a and CytC1-1 are key components of the mitochondrial electron transport chain (ETC), the expression changes in these two critical genes in the *wp3* mutant suggest that loss of WP3 function may disrupt proper assembly of the mitochondrial ETC and thereby give rise to aberrant mitochondria. We further investigated the mRNA levels of chloroplast-associated genes in seedlings of the wild type and *WP3* mutant plants. Among the six photosynthesis associated genes examined, the expression of *RbcL, RbcS*, and *CAB*, in which *RbcL* and *RbcS* encode the large and small subunits of chloroplast ribulose bisphosphate carboxylase, respectively, and *CAB* encode Chlorophyll a–b binding protein, was significantly reduced in albino sectors of *wp3* mutant leaves compared with wild type plants, while the expression of other three genes (*RPL23*, *OsSigA*, and *OsFtsZ*) was not affected (**Figure 8B**). Surprisingly, these chloroplast-associated genes display normal expression levels in green sectors of *wp3* mutant leaves (**Figure 8B**). Together, these data demonstrate that disrupted expression of *WP3* affects both mitochondrial and chloroplastic protein expression, thus suggesting that WP3 acts to coordinate the development of mitochondria and chloroplasts.

## Discussion

Chloroplasts and mitochondria are important organelles in plant cells that play ubiquitous roles in processes of global significance, including photosynthesis, amino acid biosynthesis, and ATP production. Proteomic studies have shown that chloroplasts and mitochondria contain up to several thousand gene products, including as many as 2,000–3,000 proteins in the chloroplast and ∼1,000 proteins in the mitochondrion (Schmidt et al., 2010; van Wijk and Baginsky, 2011). Among these proteins, fewer than 100, however, are encoded by the chloroplast and mitochondrial genomes, and all the rest are supplied by genes in the plant nuclear genome (Unseld et al., 1997; Sato et al., 1999). Identification of nuclear genes that are involved in the development of these organelles will therefore contribute greatly to our understanding of the coordination between the nucleus, chloroplast, and mitochondrion. Variegated plant mutants are defective in chloroplast developmental processes and mostly arise from mutations in nuclear or organellar genes. Thus, variegated mutants provide opportunities to explore the mechanisms by which the mitochondrion, the chloroplast, and the nucleus are tightly coordinated. In this report, we describe a rice variegated mutant, *wp3*, that exhibits white panicles and white-striped leaves. Using a map-based cloning approach, we isolated the mutant gene responsible for causing variegation in the leaves and panicles. Moreover, we showed that failed expression of *WP3* leads to severe developmental defects in both mitochondria and chloroplasts. Using microscopy and biochemical analyses, we were able to demonstrate that *WP3* encodes a protein that localizes to the mitochondria. Based on these observations, we conclude that *WP3* is a nuclear genome-encoded mitochondrial protein that plays critical roles during mitochondrial development. Because WP3 was not detected in chloroplasts in our study, we further propose that the chloroplast defects observed in the rice *wp3* mutant are most likely due to a lack of properly developed mitochondria. This is consistent with the previous finding in maize NCS mutants that damaged mitochondria also caused chloroplast developmental defects by distinct mechanisms.

In our previous study, the white-stripe phenotype in *wp3* mutant leaves was thoroughly described but was not analyzed genetically (Li et al., 2003). It seems that white-striped leaves are closely associated with white panicles in this mutant. In the current study, however, we show that white-striped leaves in the *wp3* mutant are maternally inherited, which is distinct from white panicles that are inherited in a typical Mendelian manner. The maternal inheritance of white-striped leaves was first demonstrated by genetic analysis in reciprocal crosses between the *wp3* mutant and the wild type TP309. White striped leaves were only observed in F_1_ plants when *wp3* was the maternal parent, but not in crosses in which *wp3* was the paternal parent. Moreover, in our complementation test, we observed that the T_0_ plants all exhibited white stripes, although their white-panicle phenotypes were all rescued. We thus surmise that the leaf stripes in T_0_ plants could result from chloroplast defects in the original plant materials that were used as the receptor in the complementation test. Indeed, we observed the rescue of the white-stripe phenotype in the subsequent T_2_ and T_3_ generations. To explain these two distinct modes of inheritance of white panicles and white-striped leaves in the *wp3* mutant, we propose the following model based on the results of our study. Failed expression of *WP3* in the rice *wp3* mutant first leads to mitochondrial developmental defects in the panicles, and these mitochondrial defects then cause a dramatic reduction in the numbers of functional chloroplast in panicles, and very likely in ovary cells as well. In next-generation seedlings, only very few chloroplasts are inherited maternally from the mutant plants, which then results in missing chloroplasts in some cells because they physically segregate during cell division. Next, these cells without chloroplasts continuously grow, divide, and eventually form the white stripes in the leaves.

Mitochondria are mobile, dynamic organelles that constantly fuse and divide. Fine regulation of these processes is critical for mitochondrial inheritance and functional maintenance (Westermann, 2010). Here, we found out that mitochondria in the *wp3* rice mutant show severe defects in the dynamic morphological change process as they progress to maturation. In the *wp3* mutant, a large number of small round mitochondria were observed, and these mitochondria could not further develop into mature mitochondria that are elongated in shape. To explain these unusual changes, we speculate that the small mitochondria in *wp3* mutant plants possibly result from reduced mitochondrial fusion or accelerated mitochondrial division. Considering that the WP3-GFP fusion protein localizes to mitochondria, it is very likely that rice WP3 is targeted to the mitochondria and has essential roles in mitochondrial dynamics, including both fusion and fission, and that loss of WP3 will cause defects in these critical processes. Since *WP3* expression is mainly detected in young rice panicles during heading and not in other green tissues, we propose that the WP3-mediated regulation of mitochondrial function is mainly restricted to heading panicles in rice. Most likely, there is a particularly pronounced need for the WP3 protein for mitochondrial function during early, rapid cell proliferation stages of panicle development. Thus, *WP3* provides us with the novel insight that plant cells from distinct organs or tissues can have unique regulators to modulate the development of their mitochondria.

Because *WP3* was shown to play critical roles in chloroplast and mitochondria development in the rice panicle, we then hypothesized that its orthologs in other plant species may have a similar function. However, sequence analysis only identified *WP3* orthologs in the genomes of wild rice, sorghum, and foxtail, suggesting similar functions for *WP3* in these species; no *WP3* orthologs were identified in *B. distachyon*, maize, Arabidopsis, and poplar. It is possible that the function of WP3 could be replaced by other genes or pathways in those species that lack an identifiable ortholog of *WP3*. Alternatively, the failure to identify *WP3* orthologs in some species might be a result of the relative incompleteness of the current genome assembly, such as in maize.

Leaves and panicles are two physiologically different organs in the grasses. The leaf is enriched in chloroplasts, and is where photosynthesis specifically occurs, whereas the panicle is an organ involved in reproduction. Because fewer chloroplasts develop in panicle cells, chloroplasts seem to be less functional in this specific organ compared to leaves. In line with this, the role of plastids changes quickly at the heading stage when panicles are generated (Inaba and Ito-Inaba, 2010). Thus, there must be a huge difference in plastid development between the leaf and panicle, and monocot plants may have developed some precisely controlled mechanisms to regulate chloroplast functions in their panicles. At present, little information is available about chloroplast modulation during panicle development in monocots, although many chloroplast defective mutants have been extensively studied. In this report, we identified a novel chloroplast defective mutant in rice. Unlike those mutants that mainly exhibit aberrant leaf color, such as *virescent* (*v*), *stripe* (*st*), *albino, chlorina, zebra*, and *yellow variegated, wp3* exhibits both white panicles and white-striped leaves on individual plants. Notably, the *WP3* gene was not transcribed in leaves, but intriguingly, was expressed exclusively in young panicles. Loss of *WP3* expression caused defective chloroplast development in panicles, indicating that *WP3* is a specific regulator of plastid development in this organ. Based on these findings, we conclude that the *wp3* mutant is an ideal model to explore the mechanisms by which chloroplast development is specifically regulated in rice panicles. It is interesting, as mentioned above, that the two previously known white panicle mutants, *wp1* and *wp2*, have similar phenotypes to *wp3*, but that their respective genetic loci are different from *wp3* (Sanchez and Khush, 1994, 2000). Therefore, further identification and eventual cloning of *WP1* and *WP2* and elucidation of the functional connections between *WP1*, *WP2*, and *WP3* will undoubtedly improve our understanding of how plastid development is specifically regulated in the panicle of monocotyledonous plants.

## Materials and Methods

### Plant Materials

*White panicle3* (*wp3*) is a naturally occurring mutant of rice (*Oryza sativa* L.) derived from the F_6_ progeny of a cross between the inbred lines ‘Zhefu802’ × ‘Xiushui11’ (Li et al., 2003). F_2_ populations used for gene mapping were developed by crossing *wp3* to a *japonica* cultivar, ‘Zhi7.’ All materials were planted in Beijing during the summer, and were grown on Hainan Island in southern China in the winter.

### Primers

The oligonucleotide primers used in this study are listed in **Supplementary Table S2**.

### Microscopy

Three-week-old plant leaves and heading panicles were harvested for histological analysis and transmission electron microscopy. Root tips from 1-week-old plants were used for fluorescence microscopy. At least 20 cells in each section were examined and photographed for each group in the experiment.

For histological analysis, leaf and panicle samples were cut into 1 mm × 3 mm pieces, fixed in 2.5% glutaraldehyde together with 2% osmium tetroxide, and embedded in Spurr’s resin. Transverse sections were prepared and stained with 1% toluidine blue solution. Light micrographs were taken using an Olympus BH-2 microscope. Mitotracker Red (Genmed Gene) staining was performed according to the manufacturer’s instructions. Fluorescence microscopy was performed on a Leica TCS SP5 confocal system.

For TEM analysis, leaf and panicle samples were soaked in primary fixation buffer (2.5% glutaraldehyde in 100 mM cacodylate buffer, pH 7.4) and post-fixed for 2 h in secondary fixation buffer (1% OsO_4_ in 100 mM cacodylate buffer, pH 7.4). The fixed samples were dehydrated in an ethanol series, embedded in resin, and stained by uranyl acetate together with lead citrate. Ultrathin sections were observed by TEM (JOEL JEM-1010 electron microscope) as described previously (Tamura et al., 2003).

### Measurement of Photosynthetic Pigment Concentration

Pigments were extracted from equal fresh weights of leaf and panicle tissues with 80% ice-cold acetone. The concentrations of chlorophylls and carotenoids were determined with a UV/VIS spectrophotometer according to a previous report (Ronen et al., 1999).

### Positional Gene Cloning

F_2_ progeny plants with white panicles (which were from a cross between *wp3* and ‘Zhi7’) were used for genetic mapping. Fine-scale mapping was performed using markers developed by comparing the genomic sequences of the *indica* cultivar 93-11^2^, and the *japonica* cultivar^3^ ‘Nipponbare.’ The DNA sequences of the PCR primers used to amplify these markers are given in **Supplementary Table S2**.

### Genome Sequence Analysis

The *japonica* genome sequence and annotation were downloaded from the Rice Genome Annotation Project, release 6. The *O. brachyantha* sequence was provided by M. Chen. The sorghum, *B. distachyon*, foxtail millet, and maize genome sequences were downloaded from Phytozome v7.0^4^. The RepeatMasker program was used for transposable element annotation. The Fgenesh program was used for gene annotation with subsequent manual inspections. ClustalW was used for multiple sequence alignment with manual inspections. HMMER software (version 2.3.2) was used to construct an HMM profile.

### DNA Constructs and Rice Transformation

For the functional complementation test, the 5.5 kb genomic DNA fragment containing the entire *WP3* coding region and the 3-kb upstream and 2-kb downstream sequences was obtained as an *Eco*R I – *Sal* I double digestion fragment from the IRBB56 BAC clone P0552C05, and was inserted into the binary vector pCAMBIA1300^5^ to generate pCAMBIA1300WP3. The empty pCAMBIA1300 vector was used as a negative control. pCAMBIA1300WP3 was used to transform callus derived from the homozygous *wp3* mutant seeds *via* agrobacterium-mediated transformation as described previously (Xiao et al., 2009).

To generate the WP3 overexpression construct, gene specific primers, 5′-CCC*g-gatcc*GTCGGAATCACCGATAAG-3′ and 5′-CCC*gagctc*TCAGGGATG-GCGGCCACC-3′ (BamHI and SacI sites are italicized in lower case letters) were used to amplify the WP3 coding sequence from rice genomic DNA. The PCR fragment was confirmed by sequencing and then cloned into the vector pTCK303 (Hiei et al., 1994) to create the WP3 overexpression vector in which the gene is driven by a maize ubiquitin promoter.

For construction of the WP3-GFP expression vector, gene-specific primers (**Supplementary Table S2**) were used to amplify the coding sequence of *WP3* using genomic DNA as template. We used overlapping PCR to fuse the gene fragment with the GFP encoding sequence that was amplified by PCR from the CaMV35S-sGFP(S65T)-NOS3’ KS vector (Niwa et al., 1999). The sequence of the fused WP3-GFP DNA fragment was confirmed by DNA sequencing and cloned into the pCAMBIA1300 vector. The construct was then transformed into wild type rice (cv. ‘Nipponbare’).

### RT-PCR Analysis

Total RNA was isolated from various tissues of the *wp3* mutant and wild-type plants using the RNeasy Plant Mini kit (Qiagen) according to the manufacturer’s instructions. For each sample, first-strand cDNA was synthesized from 2 μg total RNA primed with oligo(dT) using M-MLV reverse transcriptase (Promega) in a 25 μl reaction volume. A 0.5 μl sample of each reverse transcription reaction was then used as a template in the subsequent PCR amplifications. The primer sequences are listed in **Supplementary Table S2**. PCR was performed for 25 cycles (for ACTIN 1) or 30 cycles (for *WP3*). PCR reaction conditions were as follows: an initial denaturation at 94°C for 5 min, followed by 30 cycles of 94°C for 30 s, 58°C for 30 s, and 72°C for 50 s, with a final 5 min extension at 72°C. At least three PCR replicates per sample and three biological replicates were performed. The representative results are shown in the Figures.

### GUS Staining

The promoter region of the *WP3* gene (∼1.8 kb upstream of the ATG) was amplified from genomic DNA of ‘Nipponbare’ using the primers 5′-GTGTGCAAAACATAGCTCC-3′ and 5′-TGCAGGGGTTGCTCAAGCA-3′. The DNA fragment was then cloned into the pCAMBIA1301 vector (CAMBIA, Australia), resulting in a fusion of the promoter with the GUS reporter gene. The construct was then transformed into wild type rice (‘Nipponbare’). GUS staining was performed using the method of (Jefferson, 1989). Briefly, various tissues from ProWP3:GUS transgenic seedlings were vacuum treated for 5 min and incubated in a solution [50 mM NaP buffer, pH 7.0, 5 mM K_3_Fe(CN)_6_, 5 mM K_4_Fe(CN)_6_, 0.1% Triton X-100, and 1 mM *X*-Gluc] for 12 h at 37°C. All samples were rinsed in 75% ethanol and observed under microscope.

### Mitochondria Isolation and Immunoblot Analysis

Whole lysates of rice leaf cells were isolated using the Plant Cell Lysis/Extraction Reagent (Sigma) according to the manufacturer’s instructions. Mitochondria were isolated using the plant mitochondria isolation kit (Genmed Gene, GMS10048.3). Briefly, rice shoots were harvested and cut into 5–10 mm lengths. Cut shoots (100 g) were ground in the homogenization solution provided by the kit. The homogenate was filtered through four layers of Miracloth and was centrifuged for 5 min at 1000 × *g* to pellet the nuclei, chloroplasts, and cell debris. The supernatant was again centrifuged at 20,000 × *g* for 20 min to pellet the mitochondria. The resulting organelle pellet was washed with wash buffer twice and was then layered onto a 0–4.4% Percoll gradient and centrifuged at 30,000 × *g* for 45 min. The mitochondria formed a discrete band near the bottom of the gradient. After removing the upper plastid material, the mitochondrial layer was removed by aspiration and concentrated by washing and centrifugation at 20,000 × *g* for 15 min. Approximately 3–6 mg of total rice mitochondrial protein was obtained by this approach. Immunoblotting analysis was carried out as described by Kusumi et al. (2004). Antibodies against AOX and GFP were purchased from Agrisera and Santa Cruz, respectively. The anti-SPBase antibody was developed in our laboratory. Briefly, a peptide consisting of a 13 aa sequence of the SBPase protein (NYYVKEKYTLRYT) was synthesized and used to immunize rabbits. After the antibodies were affinity purified, immunoblotting was performed to confirm that they specifically recognize the target protein in rice chloroplast extracts.

## Author Contributions

HL and LZ conceived and designed the experiments. HL, GJ, YW, XZ, JX, and DL performed the experiments. HL, GJ, QQ, GL, MC, S, WZ, and LZ analyzed the data. HL, GJ, and LZ wrote the paper.

## Conflict of Interest Statement

The authors declare that the research was conducted in the absence of any commercial or financial relationships that could be construed as a potential conflict of interest.

## Footnotes

1 http://rapdb.dna.affrc.go.jp/

2 http://www.plantgdb.org/OsGDB/

3 http://rapdb.dna.affrc.go.jp/

4 http://www.phytozome.net/

5 http://www.cambia.org

## Supplementary Material

FIGURE S1 Ultrastructure of chloroplast in panicle branches of wild type and *wp3* plant. **(A,B)** Wild-type **(A)** and *wp3* **(B)** chloroplast in branch cell were indicated. G, grana; S, starch granule; TM, thylakoid membranes; scale bars = 0.5 μm.

FIGURE S2 Retrotransposon within *WP3* gene in *wp3* mutant. **(1)** Schematic of retrotransposon isolated from *wp3* mutant. **(2)** Sequence of the retrotransposon. FS, flanking sequence (green); TSR, target site repeat region (red); LTR, long terminal repeat (blue); PPT, polypurine tract (pink).

FIGURE S3 Amplification of *WP3* promoter in various rice varieties. PCR amplification of the *WP3* promoter region in 20 rice varieties. M indicated the DNA ladder. The experiments were performed in three biological replicates and the representative results were shown.

FIGURE S4 PCR amplification of *HygR* (*hygromycin B phosphotransferase*) and *WP3* in transgenic plants. **(A,B)** PCR amplification product of the *HygR* **(A)** and *WP3* **(B)** in total genomic DNA from 10 transformants was shown. M indicated the DNA ladder. The experiments were performed in three biological replicates and the representative results were shown.

FIGURE S5 Over-expression of *WP3* in a *japonica* rice, TP309, gives rise to darker green seedlings. 14-days seedling of TP309 and two *WP3* over-expression lines, *WP3*-OVER-3 and *WP3*-OVER-8, were shown.

TABLE S1 Pigment assessment in wild type and *wp3* mutant rice plants.

TABLE S2 Oligonucleotide primers used in the current study.
