# Supplementary figures and images for: WHITE PANICLE3, a Novel Nucleus-Encoded Mitochondrial Protein, Is Essential for Proper Development and Maintenance of Chloroplasts and Mitochondria in Rice
Source: Front Plant Sci. 2018 Jun 6;9:762. doi: 10.3389/fpls.2018.00762 (PMC5997807; doi:10.3389/fpls.2018.00762)

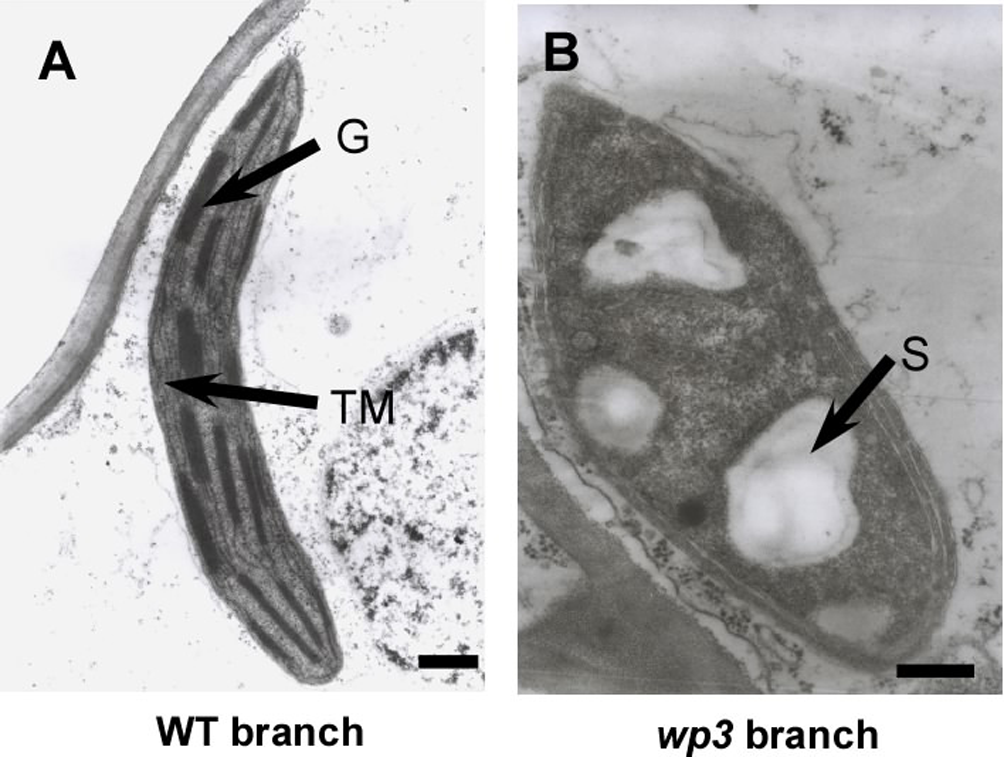

Supplement: FIGURE S1 — Ultrastructure of chloroplast in panicle branches of wild type and wp3 plant. (A,B) Wild-type (A) and wp3 (B) chloroplast in branch cell were indicated. G, grana; S, starch granule; TM, thylakoid membranes; scale bars = 0.5 μm. [file Image_1.TIF]

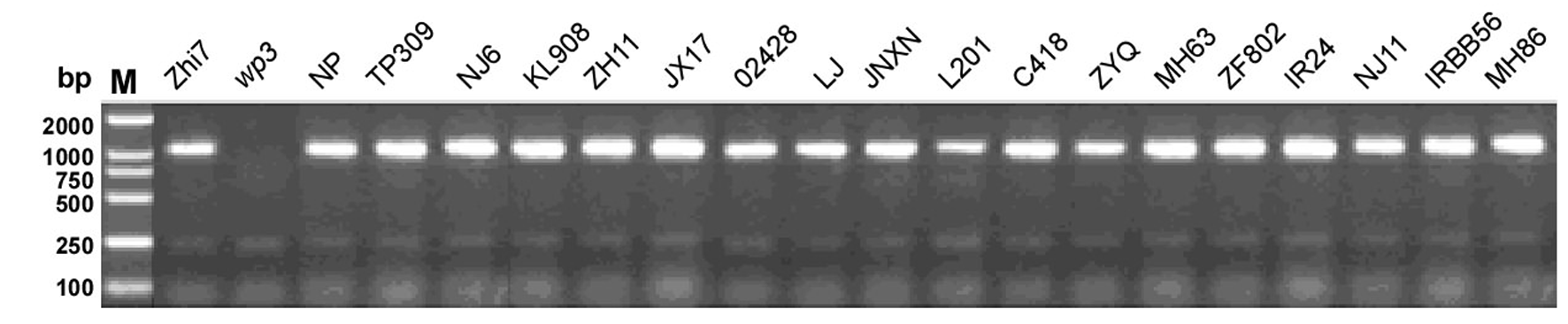

Supplement: FIGURE S3 — Amplification of WP3 promoter in various rice varieties. PCR amplification of the WP3 promoter region in 20 rice varieties. M indicated the DNA ladder. The experiments were performed in three biological replicates and the representative results were shown. [file Image_3.TIF]

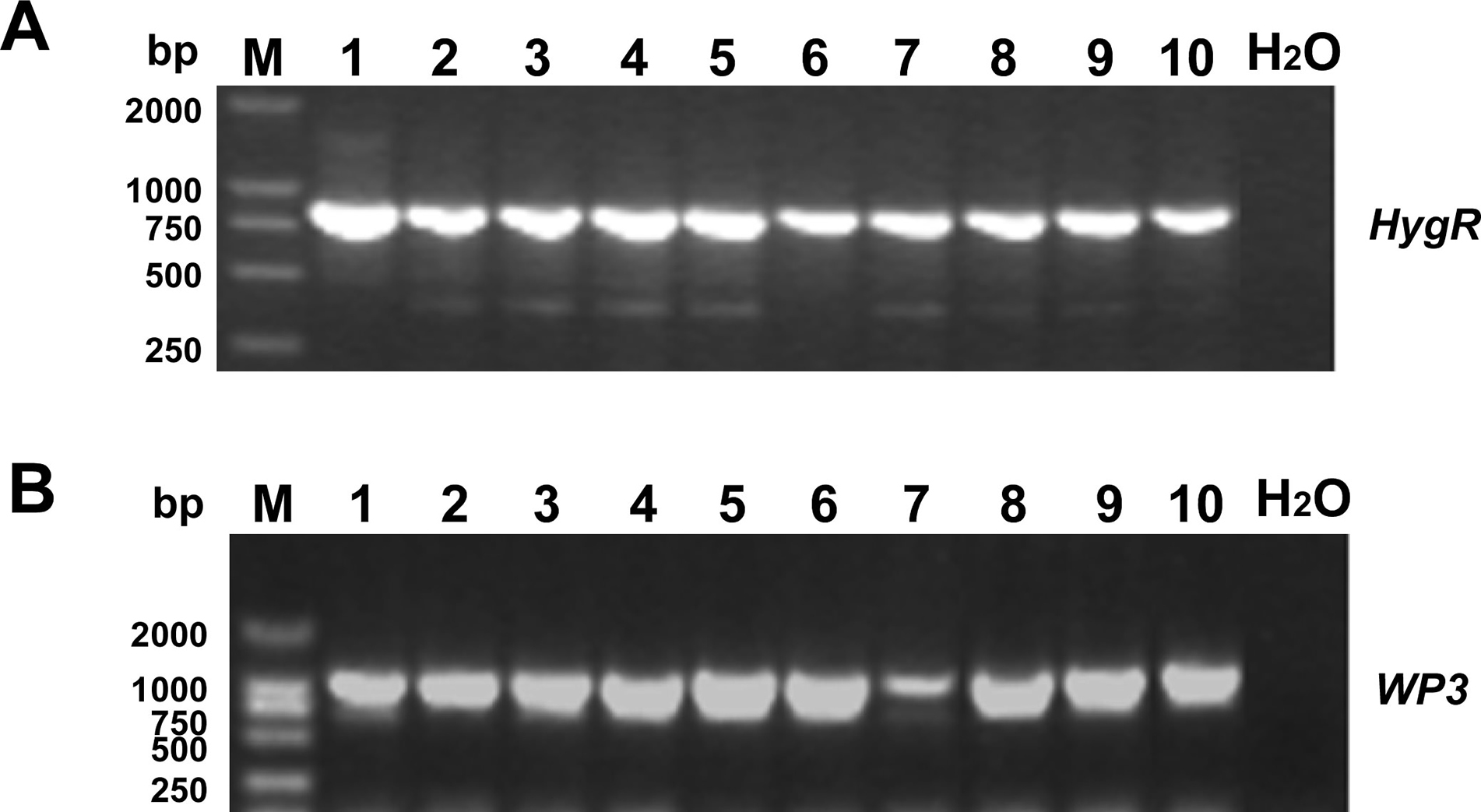

Supplement: FIGURE S4 — PCR amplification of HygR (hygromycin B phosphotransferase) and WP3 in transgenic plants. (A,B) PCR amplification product of the HygR (A) and WP3 (B) in total genomic DNA from 10 transformants was shown. M indicated the DNA ladder. The experiments were performed in three biological replicates and the representative results were shown. [file Image_4.TIF]

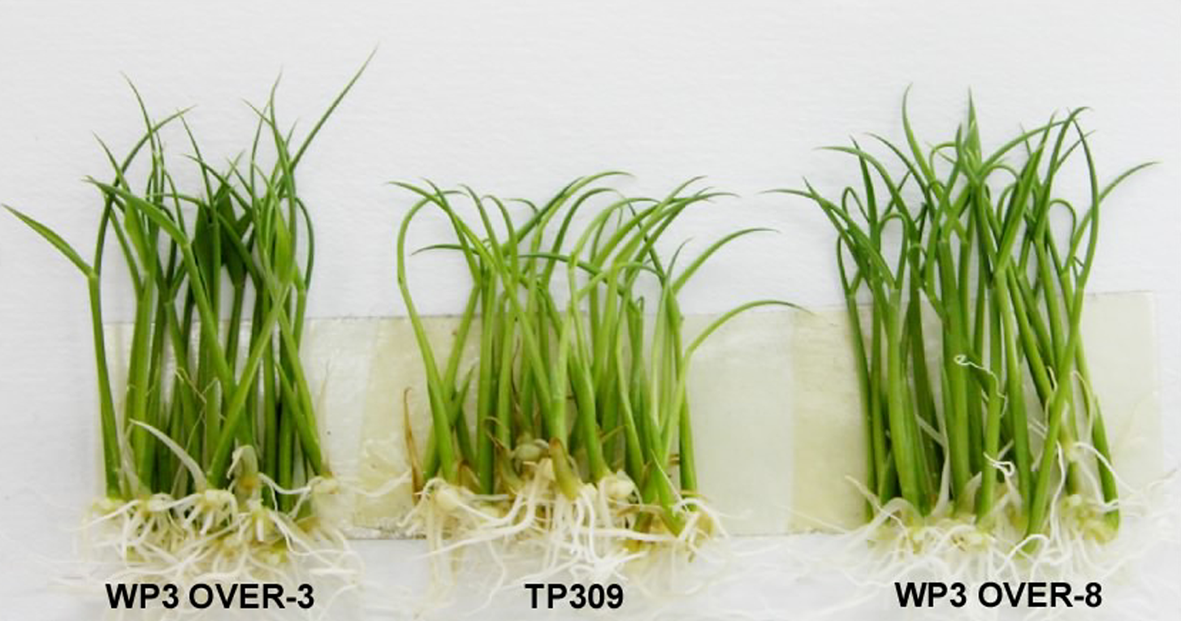

Supplement: FIGURE S5 — Over-expression of WP3 in a japonica rice, TP309, gives rise to darker green seedlings. 14-days seedling of TP309 and two WP3 over-expression lines, WP3-OVER-3 and WP3-OVER-8, were shown. [file Image_5.TIF]
