# Supplementary material for: WHITE PANICLE3, a Novel Nucleus-Encoded Mitochondrial Protein, Is Essential for Proper Development and Maintenance of Chloroplasts and Mitochondria in Rice
Source: Front Plant Sci. 2018 Jun 6;9:762. doi: 10.3389/fpls.2018.00762 (PMC5997807; doi:10.3389/fpls.2018.00762)
Supplement: FIGURE S2 — Retrotransposon within WP3 gene in wp3 mutant. (1) Schematic of retrotransposon isolated from wp3 mutant. (2) Sequence of the retrotransposon. FS, flanking sequence (green); TSR, target site repeat region (red); LTR, long terminal repeat (blue); PPT, polypurine tract (pink). [file Image_2.PDF]

1, Schematic of retrotansposon isolated from *wp3* mutant:

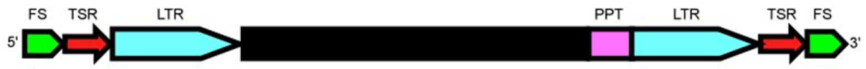

2, Sequence of the retrotransposon. FS, flanking sequence (green); TSR, target site repeat region (red); LTR, long terminal repeat (blue); PPT, polypurine tract (pink):

TGAGTGTTCCTTGACAACTACCAAAACAATATTGTCTTGTCCTCATCTGGTACCCTAAAACCTTTGATA
CACCGATTCCCTCACCACTGATCTGCTGCTAGCCACTGAGTGTCGGAATCACCGATAAGTACTAACTGT
GATAAAATATGAAGCGTTAAACCCTTTACCTCGGGCCGCACCTTTGTTAAGTAGTGGTAGAAACAGCCT
CTACCGTGGCTTACAATATTCAGTTACAAGTGATAAGAAGATCAAACAGATTCGGAGATAGAAATCTAA
TCCTAACAACCGGATGAAGTAGCTGCGTCATTATCGCAAGCTGCATGCACCTAATCTGGGCGAGGTAG
CTGTGTTGTAGTGCATGTTGCATGCATGCACGTACATATCATTTCACAGCCCCTCTCAATCATAACCTGG
TCAGGTTAAGATTGTGTTTGAAATGTTCCAGAAGGCGAACTGACAAAGCTTTTGTAAAACCATCAGCA
ACCTGATCTCCTGAAGGAACAAAATCAATCTCTAACAATTTCTGTGATACACGCTCTCTTACAAAGTGA
TAATCAACTTCTATATGTTTAGTCCTTGCATGAAACACAGGATTAGCTGATAAATATTTGGCTCCCAAAT
TGTCACACCAAATTTTAGCAGCTTTAGGTGAATCAATTCCCAACTCTCTGAGTAAGGTCTGAACCCATA
TCAATTCAGCTGTAGTATTTGCTACAGCCTTATATTCTGCTTCTGTACTTGACCTTGACACAGTAGGTTG
CTTCCTTGCACTCCATGACACAAGATTAGAACCCAGAAAAACAGCAAATCCTCCTGTTGATTTTCTATC
ATCTACGCTACCTGCCCAGTCTGCATCTGAATATCCATGAACAAGAGTAGAAGCACTCTTATGTATATTA
AGTCCTAGGCTTGTGCACTGGTTTAGGTATCTAAGGATCCTTTTCACTGCTGTCCAATGATCAGTTGTA
GGAGCATGAAGGAACTGACAAACCTTATTTACTGAATAAGCTATATCAGGCCTAGTCAGAGTCAGATAT
TGTAGAGCACCAACTATACTTCTATATTGAGTTGCATCATTAGGTCCCAAGGGCGATCCTTCATACAGAG
TTAATTTTTCACTAACAGACAGCGGAGTACTAACTGGCTTGCAGTTTGACATGTTAACTCTCTTTAATA
AATCATTAGCATACTTTTCCTGAGTCAAAACAATACCATTGGGTACTTTAGTTACCTCAATTCCAAGAAA
GTAGTGTAGATCACCCAAATCCTTGAGTGCAAACTCTTTGTTTAAATCCTTCAGAAGTGCTGTAGTTGC
CTTCTCTGTAGAACTAGCTATAATTATATCATCAACATACACCAATACAAACATAATTATCCCTCCTTTATT
AAGAAAGAATAATGAGGTGCCAGCCTTTGAAGCTTCAAAGCCAAGTTCAACAAGTTTCTTACTTAGCC
TGGAATACCATGCTCTTGGTGCCTGTTTTAACCCATACAATGCCTTGTCCAATTTGCATACATAATTTGG
TTTAGAGAATGACTCAAAACCAGGGGGTTGCTGCATGTAGACTTCTTCTTCTAAGAATCCATGAAGAA
AGGCATTTTGAACATCTAGCTGTCTAAGACTCCAACCTCTAGAAACAGCAATAGACAGAATAATCCTAA
TAGTAGCAGCTCTAACAACAGGACTAAAGGTATCCTCATAGTCAATACCATATCTCTGTTTAAACCCCTT
TGCTACAAGTCTAGCTTTGTATCTGTCAAGTGTTCCATCTGCCTTCCTTTTAATCTTATATACCCATTTAC
ACCCTATAACATTTTGTCCTTTCTCATACGGAACTAAATACCATGTATCATTTTTTATTAAAGCATCATATT
CAGCTTCCATAGCAGCTTTCCAGTTTTTATCTCCTAGAGCCTCTTTATCATTTTGAGGTTCACCAGTAGA
AGTAAAACAACCATATTTAACAGTGCCATCAGTATATACCTTCTCTTTTCGAATACCACTCCGCAATCGA
GTGTGTGGTCTTGGTGCAGGAGCTGCCGTCGCATCACCTGTAGCTGTCGTCGCATCATCACCTGTAGCT
GTCGTCTCAGGAGGAGAGGGCTGCACCGGCTGGGACTCTGCGCCACTAGCTTCTGCGCCACTGACGC
TGTCCCCTCCAGTACCAGAAGCTGCGTCGCTGCTGCCAGCTTCCTGGTCTGTTGCAGTACTGCTGTCA
GCGTGAGGATATGACTCCAGCACTGTTCCTGATGGAACTTCTTCATGCAATACGTTCTCCACTTCCCGT
TCATGTGCTGTACCATTTTCTCCAGAAACAGCATCAGTAGCATCAGAAATAATTATATTGTCACTAGGTA
TTGGAGTATTAGCCACTGGTGCAACTACATGTGTCCCCCCTGTACTAGTCGTCTCATAATTTGTCAAGG

AGGATGGCAGTAAGAGAATTTCAGACCTAAGTCTTGCTCCTGCATTTGAGAGGAGTGTGGAGAAGGG
AAAAATATTTTCATCAAAGACAACATCCCTTGAGATATAGACACGGCCTGATGACACATCAAGGCACTT
AAACCCTTTGTGGTGAGTGCTAAAACCTAGGAAAACGCACTGTTTGGAGCGAAACTGGAGTTTGTGA
GTATTATAAGGGCGAAGATGAGGCCAACAAGCACAACCAAACACTCTCAAAGACAAGTAGTCAGGTT
TTTGGTTAAACAATTTTTCTAGAGGTGTAGAATTTTGGATGGTTTTACTGGGTACACGATTGATGAGATA
AGTGGCTGCCACAAAGACTTCATCCCAAAACTTAAGAGGCATTGATGCATAAGATAGAAGAGAGAGA
CCCACTTCAATAATATGTCGGTGTTTTCTCTCAGCTGACCCATTCTGTTGATGTGTGTGCGGGCATGACA
CATGATGATCTATTCCTATTTTTGCAAAAAAGGAATTAAGTTTTTGATATTCTCCCCCCCAATCAGTCTG
CATGGCAATAATTTTTCTATCAAACATTCTTTCAACTAAAGCCTGAAATTCTTTAAACTTCTCAAAGACC
TCAGACTTATATTTTAGCAAGTATATCCAAGTAAACTTACTAAAATCATCAATAAAACTCACATAATATTT
ATTTCTCCCAACAGACTCTGGAGCAGGGCCCCAAACATCAGAAAACACAAGTTCAAGGGGAAATTGA
GACACACTAGTAGACCTGACATAAGGTAACTGACGACTCTTTGCTTCCTGACAAGCATTACACACAGA
CTGATTGACTGACTCAACTGAACACGGGAGATTATTTTTGCTAATGACTTGCTTAACAATAGGAAGAGA
TGGATGTCCTAGACGACTATGCCACCTGGACAAGGATAGCTTGGTGGTGCCAAGTGCTTGTTTGCTGG
TTGTTCGGCCAAAGGACTTAGGGATTGGGTAGAGGCCATGCCTACATCTTCCTTCAAGCAGAATGTCC
TTCGTTACCTGATCCTTTATAGAAAAGAATTTCGAGTGAAGTTCAAGGAATGCGGAATTATCAGTGGCT
AACTGACTAGCAGATATTAGACTCTTTTTGGCTTGAGGAACATAAAGAACATTATTTAAATGAATGTTA
CGACTAGGGGTATGCACAGTAGTATGGCCAATGTGACTAATATCCATACCTGCTCCACTAGCCGTATGAA
TCTGCTCGTTGCCGTTGTATTTTTCCTTGGTGGTAAGCTTCTCTAACTCGCCAGTAATATGATCTGTAGC
ACCTGTATCAATATACCAATTTGTATCTATTCCATACGAGTTAGTAGCAGCAGCAGCAAGTTTTTCATCG
GCAACGTAATCCTCATCGAAGCGATACCAGCAGTCGGCTGCTGTGTGCCCGCGCTTGAAACAGACCTG
ACAGGTGGGGCGCCTGTCTTGGCCTCCTAAGCCGCGCCCGCGCCCACGAGCTTGGCCGCCACTGCGC
CCGCGCGGCGCGCCCCTGCCGCCGCTGCCGTTGGAGCTGTGGCCGCCGTTGTTGTTGCCGCCGCGTCC
GCCGCCGCCGCCGCCTCTGCCGCGATTGGCGACAAAAGCAGCTCCTCCCTGACTAGTGCTACGGTAGA
GATCAAGGAGGGTTTCAAAGTTGACGAGCTGGGAATACAGCTCCCCAACCGTGATTGGATCGGACTTG
TTACAGAGGTTGGAGACGATCGGGCTGAAGTCTTCATTTAGCCCAGCGATGATGTACTAGACAAGGTC
TTCTTCATCAAGTGGACGGCCGCCGGCAGCCATCTCATCACCAAGCGCTCGCATCTTGGCGACATACT
CGGCGATCTTCATTGTTCCTTTCTTCGTGTTGGTGAGAGCAAGTCTGGTGTTCACCGCCCTTGCTCGAG
TGCCGGTGGAGTAGAGTCCTTCAATGGCCCGCCATGCCTCAGCCGCTGTCTTGCATGTGGCAACTTGG
ATCAGCACATCCCTTGAAAGAGATGATAGGAGGTAGCCAAGAACAAGCTGGTCATTGGCATCCCAATC
TTCAAATGCCGGATTTGGCTTTGTTGTGGTCTTCCCATCTATGGTGACCGAGAGTTCGGCGTCTGGAGC
TTTCACAGCGCCGGTGAGATGACCCTGGAGTCGGGCGCCGCGGACTGCAGCACTCACTTGCGCTTTC
CATAGCGCGTGGTTGCCTTTTCCGAGTTTCTCTGAAACGGAATGGCCTTGAGAGAGGTTCACAGCTGC
CGTGCCTGAGGAAGACGACGACGCCATGGAAGGAGGTTGATAGATGCGATCTACCGAGGTGGAAAAA
GGCTCTGATACCATGTGATAAAATATGAAGCGTTAAACCCTTTACCTCGGGCCGCACCTTCGTTAAGTA
GTGGTAGAAACAGCCTCTACCGTGGCTTACAATATTCAGTTACAAGTGATAAGAAGATCAAACAGATT
CGGAGATAGAAATCTAATCCTAACAACCGGATGAAGTAGCTGCGTCATTATCGCAAGCTGCATGCACCT
AATCTGGGCGAGGTAGCTGTGCTGTAGTGCATGTTGCATGCATGCACGTACATATCATTTCACACTAAC
ACTCTAACAGCTAGCTCAGGCCGGTTTGCCTATAAAAGCTCTACGGGCACACACTTACCATGCCATCCA
CAAGTGCACAAGACCACCACCTACGACGTCTTACGCTCTTAGCTAGG
