# Supplementary material for: WHITE PANICLE3, a Novel Nucleus-Encoded Mitochondrial Protein, Is Essential for Proper Development and Maintenance of Chloroplasts and Mitochondria in Rice
Source: Front Plant Sci. 2018 Jun 6;9:762. doi: 10.3389/fpls.2018.00762 (PMC5997807; doi:10.3389/fpls.2018.00762)
Supplement: TABLE S1 — Pigment assessment in wild type and wp3 mutant rice plants. [file Table_1.DOCX]

Supplemental Table S1. Pigment assessment in wild type and *wp3* mutant rice.

| Pigment (μmol/g) | Wild type  panicle | *wp3* panicle | Wild type  leaf | White sectors of *wp3* leaf |
| --- | --- | --- | --- | --- |
| Neoxanthin | 2.77±0.32 | 0.37±0.06 | 32.87±6.40 | 0.20±0.03 |
| Violaxanthin | 5.50±0.67 | 0.61±0.15 | 40.59±2.55 | 0.27±0.06 |
| Lutein | 8.68±0.76 | 0.78±0.13 | 96.36±12.69 | 0.77±0.16 |
| β-Carotene | 1.55±0.30 | 0.05±0.02 | 64.45±10.62 | 0.33±0.04 |
| Total carotene | 18.5 | 1.81 | 234.27 | 1.57 |
| Chlorophyll a | 20.71±2.07 | 0.19±0.09 | 411.60±48.66 | 0.50±0.11 |
| Chlorophyll b | 12.33±1.68 | 0.83±0.45 | 195.93±16.55 | 0.27±0.07 |
