# Supplementary material for: WHITE PANICLE3, a Novel Nucleus-Encoded Mitochondrial Protein, Is Essential for Proper Development and Maintenance of Chloroplasts and Mitochondria in Rice
Source: Front Plant Sci. 2018 Jun 6;9:762. doi: 10.3389/fpls.2018.00762 (PMC5997807; doi:10.3389/fpls.2018.00762)
Supplement: TABLE S2 — Oligonucleotide primers used in the current study. [file Table_2.DOCX]

Supplemental Table S2. Primers used in the current study.

| **Name** | **Forward sequence (described 5’ to 3’)** | **Reverse sequence (described 5’ to 3’)** |
| --- | --- | --- |
| Mapping | | |
| MRG3551 | ATATTCAATAGCTGGTTTGTGC | GGGCCATGGGATGGCGGCCACCGTGCTG |
| SSR203 | CAGATCTTGATGGCTCCCAACT | ATATTTTTGTCACCCGCTGGAC |
| SSR101 | CGACTCCATGAAACAAACGAAC | AACTGACCCCTTGCCTGAAG |
| SSR17 | TCCAAAGATTTGACTCTGTAGA | TGAGAAACAAGCGAATAGATG |
| SSR 63.9 | CTCCGCGTTTCACCATTTCGTC | CGTCGTAGTACCCTCCCACCAA |
| P7 | ATTACGTATGTGCTGAGTCC | ATTGGTAGTCATGTATGCTG |
| P10 | AGCGTACGATATACTCCTAC | ATGAAGAGCAAGAACCACTG |
| P13 | GATATCAGCGGTTCAGATTG | ATGGTCAGCACGTGACAAGA |
| P3 | TCAACCACAAACCACTTTAG | TCAACCACAAACCACTTTAG |
| WP3-GFP vector construction | | |
| WP3GFP | GGGCCATGGCGGCGAGATCCTTGG | GGGCCATGGGATGGCGGCCACCGTGCTG |
| Transgenic plants detection | | |
| VHYG | TAGGAGGGCGTGGATATGTC | TACACAGCCATCGGTCCAGA |
| RETROFS | AGAAGCCGAGCTTGGAAGGA | CTGACATGCATATCAACTGG |
| RT-PCR | | |
| *ACTIN* | TCCATCTTGGCATCTCTCAG | TCCATCTTGGCATCTCTCAG |
| *WP3* | CATTCCTCCTCACCTCTGT | CATGTCGTGGCTGATGAACC |
| *AOX1a* | TGCGGCTGATGTCCACGTC | GGACGTTGTCGATCTTGCC |
| *CytC1-1* | CATGCATCTATTCGGCGTG | ATTGTGCCTTGCCTTCGTG |
| *COX1* | CAGCCTTAGTAGAAGTGGG | GCCCTAAGAAATGCATGGG |
| *SDH* | TATACCAACGGGCCTTTGG | CATCTCCAGTGCATGTGTG |
| *TOM40* | GTGACAATCCTTGTGCGCC | GTAATGGGGCTCGTTCGTC |
| *UCP1* | TTTGGAACGGCATCATCCC | GCTCCAATACCTTCCTGTC |
| *VDAC* | GAGCTAACACCAGGATTG | CTTGCCGGAATTGTTGAAG |
| *RBCL* | GGAGATCATATCCACGCTGG | CTAGCTATCTAGTTTATCTA |
| *RBCS* | CTAACTAACTACGTGGCTATG | CGATGCTTGATCTTAGCTTA |
| *RPL23* | TTAGTTTTTTTCTCTATCTA | GGAGGGAAAGTCGATTTATG |
| *CAB* | CCGTCAACAACAACGCCTGG | ACATCATCAACTTCTTCATC |
| *OsSigA* | GATTTGAGGAGTGGTATCTC | TGTGTTTGTGGATCTGACTC |
| *OsFtsZ* | AAAGGACATAACCTTGCAAG | AGTTTTCCTATTGAACCGTG |
